# Relationship between insulin resistance, coronary plaque, and clinical outcomes in patients with acute coronary syndromes: an analysis from the PROSPECT study

**DOI:** 10.1186/s12933-020-01207-0

**Published:** 2021-01-07

**Authors:** Serdar Farhan, Björn Redfors, Akiko Maehara, Thomas McAndrew, Ori Ben-Yehuda, Bernard De Bruyne, Roxana Mehran, Birgit Vogel, Gennaro Giustino, Patrick W. Serruys, Gary S. Mintz, Gregg W. Stone

**Affiliations:** 1 Icahn School of Medicine At Mount Sinai, The Zena and Michael A. Wiener Cardiovascular Institute, New York, NY USA; 2 Clinical Trials Center, Cardiovascular Research Foundation/ Columbia University Medical Center, 1700 Broadway, 9th Floor, New York, NY 10019 USA; 3 NewYork-Presbyterian Hospital/Columbia University Irving Medical Center, New York, NY USA; 4 Department of Cardiology, Sahlgrenska University Hospital, Gothenburg, Sweden; 5 The Cardiovascular Center, OLV Hospital, Aalst, Belgium; 6 Department of Cardiology, NUIG, National University of Ireland, Galway, Ireland; 7 Imperial College of Science, Technology and Medicine, London, UK

**Keywords:** Acute coronary syndrome, Insulin resistance, Insulin, Glucose, Culprit and non-culprit lesion events

## Abstract

**Background:**

We investigated the association of insulin resistance (IR) with coronary plaque morphology and the risk of cardiovascular events in patients enrolled in the Providing Regional Observations to Study Predictors of Events in Coronary Tree (PROSPECT) study.

**Methods:**

Patients with acute coronary syndromes (ACS) were divided based on DM status. Non-DM patients were further stratified according to homeostasis-model-assessment IR (HOMA-IR) index as insulin sensitive (IS; HOMA-IR ≤ 2), likely-IR (LIR; 2 < HOMA-IR < 5), or diabetic-IR (DIR; HOMA-IR ≥ 5). Coronary plaque characteristics were investigated by intravascular ultrasound. The primary endpoint was major adverse cardiac events (MACE); a composite of cardiac death, cardiac arrest, myocardial infarction, and rehospitalization for unstable/progressive angina.

**Results:**

Among non-diabetic patients, 109 patients (21.5%) were categorized as LIR, and 65 patients (12.8%) as DIR. Patients with DIR or DM had significantly higher rates of echolucent plaque compared with LIR and IS. In addition, DIR and DM were independently associated with increased risk of MACE compared with IS (adjusted hazard ratio [aHR] 2.29, 95% confidence interval [CI] 1.22–4.29, p = 0.01 and aHR 2.12, 95% CI 1.19–3.75, p = 0.009, respectively).

**Conclusions:**

IR is common among patients with ACS. DM and advanced but not early stages of IR are independently associated with increased risk of adverse cardiovascular events.

*Trial Registration* ClinicalTrials.gov Identifier: NCT00180466.

## Background

Diabetes mellitus (DM) is increasing in prevalence and is associated with an increased risk of adverse short and long-term cardiovascular events [1, 2]. More than 50% of deaths among patients with DM are due to cardiovascular causes [3] despite tremendous development in medical treatment of DM. DM is caused by insulin resistance (IR) and/or defective insulin secretion [4], and IR is considered the earliest stage in the development of DM [4, 5]. The extent of IR has been associated with increased risk of cardiovascular events in both non-diabetics as well as patients with DM [4, 5], and IR has been associated with an increased prevalence of lipid-rich coronary plaques and is associated with increased risk for cardiovascular events [6–8]. However, no prospective data on the association of IR with coronary plaque morphology in patients with acute coronary syndromes (ACS) are available, especially with respect to non-culprit coronary lesions. Therefore, we aimed to investigate the association between baseline IR and coronary plaque morphology and clinical outcomes in patients with ACS enrolled in the Providing Regional Observations to Study Predictors of Events in Coronary Tree (PROSPECT) study [9].

## Methods

PROSPECT (NCT00180466) was an international study (United States and Europe) that investigated the natural history of coronary atherosclerosis in a population of patients admitted for ACS and treated successfully with percutaneous coronary intervention (PCI). The study design and main results have been published elsewhere [9]. In brief, 697 patients with ACS who were treated successfully with PCI for all lesions deemed responsible for the index event were enrolled. All patients underwent angiography as well as grayscale and virtual histology (VH) intravascular ultrasound (IVUS) of the left main coronary artery as well as 6–8 cm of the proximal portion of each major epicardial coronary vessel after successful intervention of all culprit lesion(s). The present study includes patients in whom data on fasting insulin and glucose concentrations at baseline were available to determine IR. Patients were stratified according to their IR index, as defined by the homeostasis model assessment (HOMA-IR; fasting insulin [mU/mL] × fasting plasma glucose [mg/dL]/405). Patients without established DM were divided into the following groups based on their HOMA-IR values: Insulin sensitive (IS, HOMA-IR ≤ 2), likely-IR (LIR, 2 < HOMA-IR < 5), and diabetic-IR (DIR, HOMA-IR ≥ 5) [10–12]. Patients with established DM were considered a separate group.

### Intravascular imaging

Grayscale IVUS and VH-IVUS analyses were performed using QCU-CMS (Medis medical imaging systems bv, Leiden, the Netherlands), pcVH 2.1 (Volcano Corporation, San Diego, California) for contouring and data output, and proprietary software (qVH, Cardiovascular Research Foundation, New York, NY, USA) for segmental quantitative and qualitative analysis [13, 14]. The external elastic membrane and lumen borders were detected approximately every 0.4 mm (depending on heart rate) and used to determine the external elastic membrane area, lumen area, and plaque area and burden (defined as 100 × plaque area/external elastic membrane area). A non-culprit lesion was defined as ≥ 3 consecutive frames with plaque burden ≥ 40%. VH-IVUS allows the characterization of 4 different plaque components (red corresponds to necrotic core, green to fibrous tissue, light green to fibrofatty, and white to dense calcium). Based on its compositional traits, each lesion was classified as thin-cap fibroatheroma (TCFA), thick-cap fibroatheroma, fibrotic plaque, or fibrocalcific plaque [14, 15]. All IVUS frames were co-registered to the angiographic roadmap using fiduciary side branches for alignment.

### Endpoints

The primary endpoint was the incidence of major adverse cardiac events (MACE), defined as cardiac death or arrest, myocardial infarction, or rehospitalization for unstable or progressive angina. The median follow up was 3.4 years. Endpoints were adjudicated by an independent events committee using original source documents and without knowledge of other patient data. Based on the follow-up angiography, recurrent MACE was adjudicated as occurring at initially treated (culprit), previously untreated (non-culprit) lesions, or indeterminate. On the basis of principal results [9], a high-risk lesion was defined as having 2 or more of the following: Plaque burden ≥ 70%, minimum lumen area (MLA) ≤ 4.0 mm^2^, or TCFA.

### Statistical analysis

Baseline clinical and imaging data were stratified based on HOMA-IR and DM. Categorical variable are expressed as count and percentage and compared using the χ^2^ or Fisher exact test. Continuous variables are reported as median (interquartile range) and compared using Student’s *t*-test or nonparametric Wilcoxon rank sum test. Outcomes are reported as Kaplan–Meier percentage and number of events and compared using the log-rank. Logistic regression analysis, adjusted for age and sex, was used to evaluate the relationship between the HOMA-IR groups and the probability of having IVUS any features of high-risk plaque (MLA ≤ 4mm^2^, TCFA, and plaque burden ≥ 70%). A multivariable Cox proportional hazards regression model was estimated the adjusted risk of 3-year MACE associated with HOMA-IR and DM. In regard to the risk of overall MACE, the following covariates were included in the model: The presence of ≥ 1 lesion with a TCFA, presence of ≥ 1 lesion with an MLA ≤ 4 mm^2^, age, sex, use of aspirin in the preceding 7 days, and a history of PCI. Due to a smaller number of events, the adjusted risks of culprit MACE and non-culprit MACE included the following reduced covariate set: Age, sex, use of aspirin in the preceding 7 days, and a history of PCI [16]. A p value < 0.05 was considered statistically significant. All statistical tests were performed using SAS version 9.2 (SAS Institute, Cary, NC, USA).

## Results

### Baseline characteristics

Out of 697 patients enrolled in the *PROSPECT* study, 507 patients had available data on HOMA-IR and 99 (19.5%) had established DM. Among patients without DM, 109 patients (21.5%) were categorized as LIR, and 65 patients (12.8%) were categorized as DIR. The remaining 234 (46.2%) patients had normal insulin sensitivity (IS). Patient clinical characteristics are presented in Table 1. Patients with DIR and established DM had higher prevalence of metabolic risk factors compared with IS and LIR groups. Patients with DM were found to have the lowest glomerular filtration rate compared with the remaining 3 groups. With the exception of significantly elevated triglyceride concentration in patients with DM compared with the other groups, the baseline lipid profile was comparable across the groups.

**Table 1:** Baseline and clinical characteristics

	Insulin sensitive (n = 234)	Likely insulin resistance (n = 109)	Diabetic insulin resistance (n = 65)	Diabetes mellitus (n = 99)	p value
Age, years	59.3 (51.5–67.7)	57.2 (49.2–65.0)	57.6 (49.6–65.5)	60.8 (54.5–69.9)	0.056
Male	76.5 (179/234)	78.9 (86/109)	84.6 (55/65)	69.7 (69/99)	0.15
Waist circumference, cm	97.0 (89.0–103.0)	104.1 (96.0–113.0)	106.7 (96.5–115.0)	104.1 (94.0–114.3)	< 0.0001
Body mass index, kg/m^2^	26.9 (24.5–29.3)	29.6 (26.5–33.5)	30.0 (27.1–35.5)	29.4 (26.3–32.8)	< 0.0001
Systolic BP, mm Hg	130 (112–144)	130 (119–140)	133 (120–143)	130 (115–146)	0.50
Diastolic BP, mm Hg	73 (63–81)	75 (70–82)	74 (68–84)	70 (67–85)	0.19
Prior myocardial infarction	11.7 (27/231)	8.3 (9/108)	10.8 (7/65)	12.1 (12/99)	0.79
Family history of CAD	39.4 (82/208)	48.0 (47/98)	56.1 (32/57)	34.5 (29/84)	0.04
Hypertension requiring medication	42.0 (97/231)	46.8 (51/109)	46.2 (30/65)	65.3 (64/98)	0.002
Hypercholesterolemia requiring medication	44.3 (94/212)	33.3 (33/99)	39.3 (24/61)	60.0 (57/95)	0.002
Current smoking	50.4 (117/232)	41.5 (44/106)	40.0 (26/65)	40.2 (39/97)	0.18
Clinical presentation					
STEMI	26.1 (61/234)	27.5 (30/109)	26.2 (17/65)	26.3 (26/99)	0.99
Non-STEMI	67.1 (157/234)	69.7 (76/109)	73.8 (48/65)	72.7 (72/99)	0.64
Unstable angina	6.8 (16/234)	2.8 (3/109)	0.0 (0/65)	1.0 (1/99)	0.01
Laboratory parameters during index hospitalization					
eGFR, mL/min	93.9 (75.2–116.8)	104.7 (78.5–140.6)	109.6 (79.7–138.0)	97.8 (68.7–127.5)	0.02
Total cholesterol, mg/dL	170.0 (148–195)	172.0 (153.8–192.3)	171.0 (153.8–197)	161.5 (140.5–198.5)	0.66
HDL, mg/dL	38.6 (34–46)	38.6 (32–41)	38.6 (33–44)	39.0 (32–49)	0.13
LDL, mg/dL	101.2 (81.1–129.2)	104.4 (78.4–125.6)	109.0 (83–149.6)	93.6 (67–121.0)	0.12
Triglycerides, mg/dL	118.0 (88.6–163.0)	128.0 (88.6–177.1)	140.5 (101.5–179.0)	145.5 (97.0–206.0)	0.02
Fasting glucose, mg/dL	92 (87–100)	107 (96–115)	120 (106–139)	123 (103–160)	< 0.0001
Fasting insulin, µU/L	6.0 (4.0–8.0)	13.0 (11.0–16.0)	31.5 (24.0–55.0)	11.0 (6.0–22.0)	< 0.0001
HbA1c, %	5.6 (5.2–6.0)	5.7 (5.3–6.0)	5.7 (5.3–5.9)	6.6 (6.2–7.5)	< 0.0001
C-reactive protein, mg/dL	6.90 (2.10–17.00)	12.55 (3.70–27.65)	8.65 (3.40–24.00)	6.95 (2.60–21.85)	0.005
Medication at discharge					
Lipid lowering drug	88.5 (207/234)	90.8 (99/109)	90.8 (59/65)	86.9 (86/99)	0.78
Aspirin	98.3 (230/234)	97.2 (106/109)	96.9 (63/65)	98.0 (97/99)	0.88
Thienopyridine	98.3 (230/234)	100.0 (109/109)	98.5 (64/65)	97.0 (96/99)	0.37
Beta blocker	91.5 (214/234)	89.9 (98/109)	93.8 (61/65)	89.9 (89/99)	0.80

Values are median (interquartile range) or % (n/N). BP, blood pressure; CAD, coronary artery disease; eGFR, estimated glomerular filtration rate; HbA1c, glycosylated hemoglobin; HDL, high-density lipoprotein; LDL, low-density lipoprotein; STEMI, ST-segment elevation myocardial infarction

### IVUS characteristics of non-culprit lesion

IVUS parameters stratified according to HOMA-IR status are presented in Table 2. On grayscale IVUS evaluation, patients with DIR had significantly higher total lesion length compared with the established DM, LIR, and IS groups. Furthermore, patients with DIR and established DM had a higher prevalence of echolucent plaques compared to patients with IS and LIR. There was no difference in the proportion of patients with MLA ≤ 4 mm^2^ or plaque burden ≥ 70% among the HOMA-IR groups. On VH-IVUS analysis there were no significant differences in volumetric parameters or lesion phenotypes across the 4 groups.

**Table 2:** Patient level intravascular ultrasound features of non-culprit lesions

	Insulin sensitive (n = 234)	Likely insulin resistance (n = 109)	Diabetic insulin resistance (n = 65)	Diabetes mellitus (n = 99)	p value
Grayscale intravascular ultrasound					
Number of lesions	5 (3–6)	5 (4–6)	6 (4–7)	5 (4–6)	0.05
≥ 1 echolucent plaque	14.5 (32/221)	8.7 (9/103)	23.8 (15/63)	20.2 (19/94)	0.03
≥ 1 plaque rupture	12.2 (27/221)	20.4 (21/103)	17.5 (11/63)	11.7 (11/94)	0.18
Total lesion length, mm	69.3 (40.0–103.0)	72.4 (46.1–96.8)	94.5 (61.1–115.9)	66.1 (45.6–95.0)	0.02
Plaque volume, %	49.6 (47.0–52.3)	48.8 (46.1–51.2)	49.0 (46.6–52.5)	49.6 (46.8–52.3)	0.27
Average EEM CSA, mm^3^/mm	15.8 (13.8–18.4)	16.3 (14.6–19.5)	17.3 (14.3–19.2)	16.5 (14.2–18.4)	0.11
Average luminal CSA, mm^3^/mm	8.0 (6.8–9.2)	8.6 (7.1–10.1)	8.7 (7.0–9.9)	8.0 (7.0–9.4)	0.04
VH-intravascular ultrasound					
Average necrotic core CSA, mm^3^/mm	0.54 (0.26–0.82)	0.55 (0.34–0.89)	0.49 (0.34–0.73)	0.50 (0.31–0.78)	0.90
Average dense calcium CSA, mm^3^/mm	0.24 (0.11–0.42)	0.21 (0.14–0.40)	0.18 (0.11–0.34)	0.24 (0.12–0.41)	0.75
Average fibrous tissue CSA, mm^3^/mm	2.48 (1.89–3.14)	2.64 (1.99–3.44)	2.79 (2.15–3.62)	2.73 (2.10–3.37)	0.10
Average fibrofatty CSA, mm^3^/mm	0.80 (0.49–1.20)	0.82 (0.54–1.11)	0.95 (0.56–1.59)	0.92 (0.57–1.40)	0.12
Total number of VH-TCFA lesions per patients	1.0 (0.0–2.0)	1.0 (0.0–2.0)	1.0 (0.0–1.0)	1.0 (0.0,1.5)	0.43
High-risk plaque characteristics					
≥ 1 lesion with MLA ≤ 4 mm^2^	57.9 (128/221)	46.6 (48/103)	55.6 (35/63)	60.6 (57/94)	0.18
≥ 1 lesion with plaque burden ≥ 70%	29.9 (66/221)	33.0 (34/103)	39.7 (25/63)	35.1 (33/94)	0.49
≥ 1 VH-TCFA	54.2 (110/203)	63.8 (60/94)	53.3 (32/60)	55.7 (49/88)	0.43

Values are median (interquartile range) or % (n/N). CSA, cross-sectional area; EEM, external elastic membrane; MLA, minimal lumen area; TCFA, thin-cap fibroatheroma

### Association between HOMA-IR and IVUS features of high-risk plaque

HOMA-IR was not associated with the high-risk plaque features including (i) presence of ≥ 1 TCFA (LIR versus IS: Odds ratio [OR] 1.44, 95% confidence interval [CI] 0.87–2.39, p = 0.14; DIR versus IS: OR 0.94, 95% CI 0.53–1.68, p = 0.45; DM versus IS: OR 1.08, 95% CI 0.65–1.79, p = 0.94); (ii) presence of ≥ 1 lesion with MLA < 4 mm^2^ (LIR versus IS: OR 0.64, 95% CI 0.39–1.03, p = 0.051; DIR versus IS: OR 0.95, 95% CI 0.54–1.67, p = 0.80; DM versus IS: OR 1.08, 95% CI 0.65–1.77, p = 0.32) and (iii) plaque burden ≥ 70% (LIR versus IS: OR 1.19, 95% CI 0.72–1.98, p = 0.83; DIR versus IS: OR 1.56, 95% CI 0.87–2.80, p = 0.28; and DM versus IS: OR 1.28, 95% CI 0.76–2.14, p = 0.87). There were no differences in the prevalence of TCFA according to HOMA-IR status among patients non-culprit MACE (p = 0.38) those without non-culprit MACE (p = 0.77).

### Clinical outcomes

**Fig. 1:**
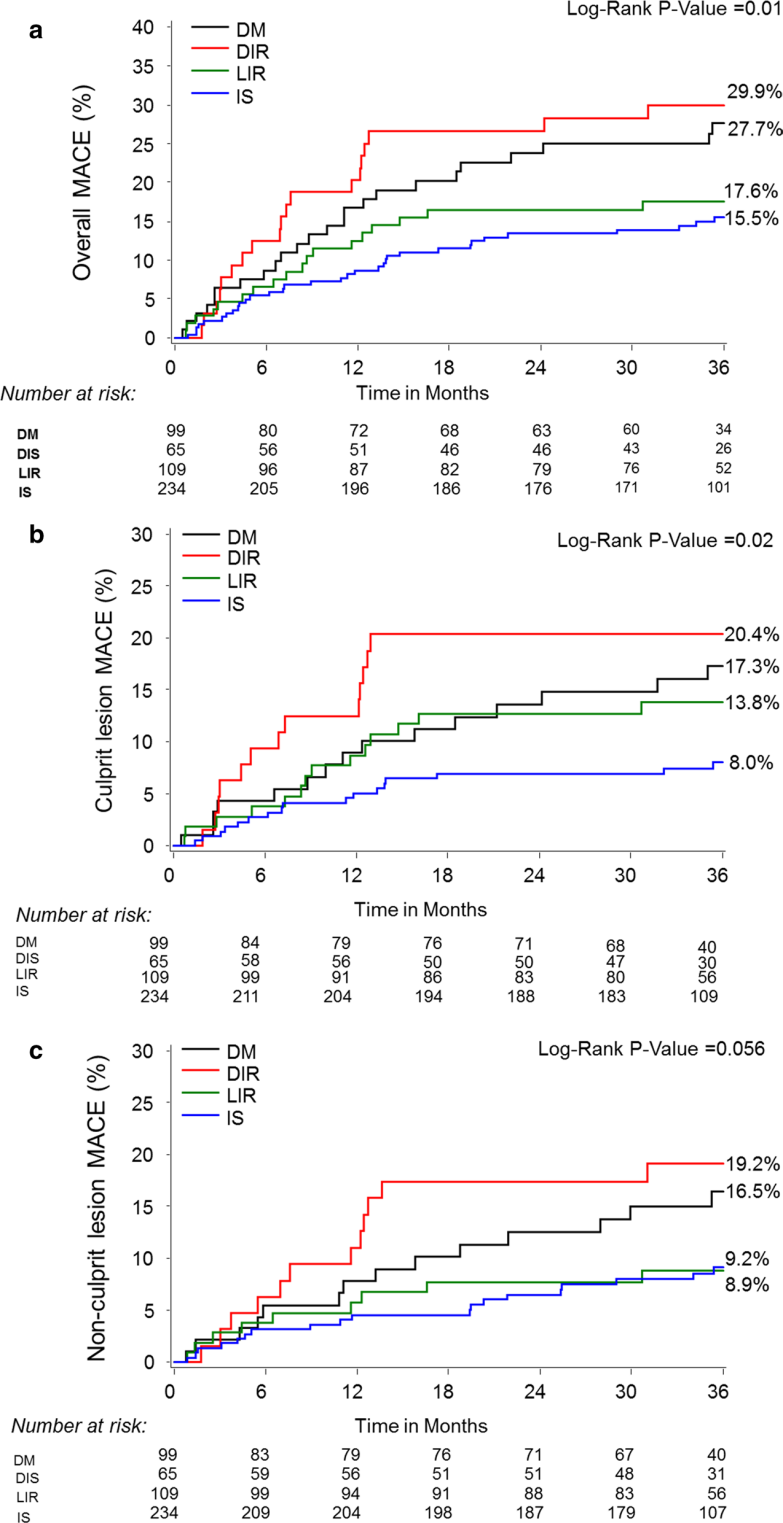
Clinical Outcomes Through 3 Years of Follow-up. **a** Overall major adverse cardiac events (MACE), **b** culprit lesion MACE, and (**c**) non-culprit lesion MACE stratified by homeostasis model assessment insulin resistance and diabetes mellitus status. DIR = diabetic insulin resistance; DM = diabetes mellitus; IS = insulin sensitive; LIR = likely insulin resistant

**Fig. 2:**
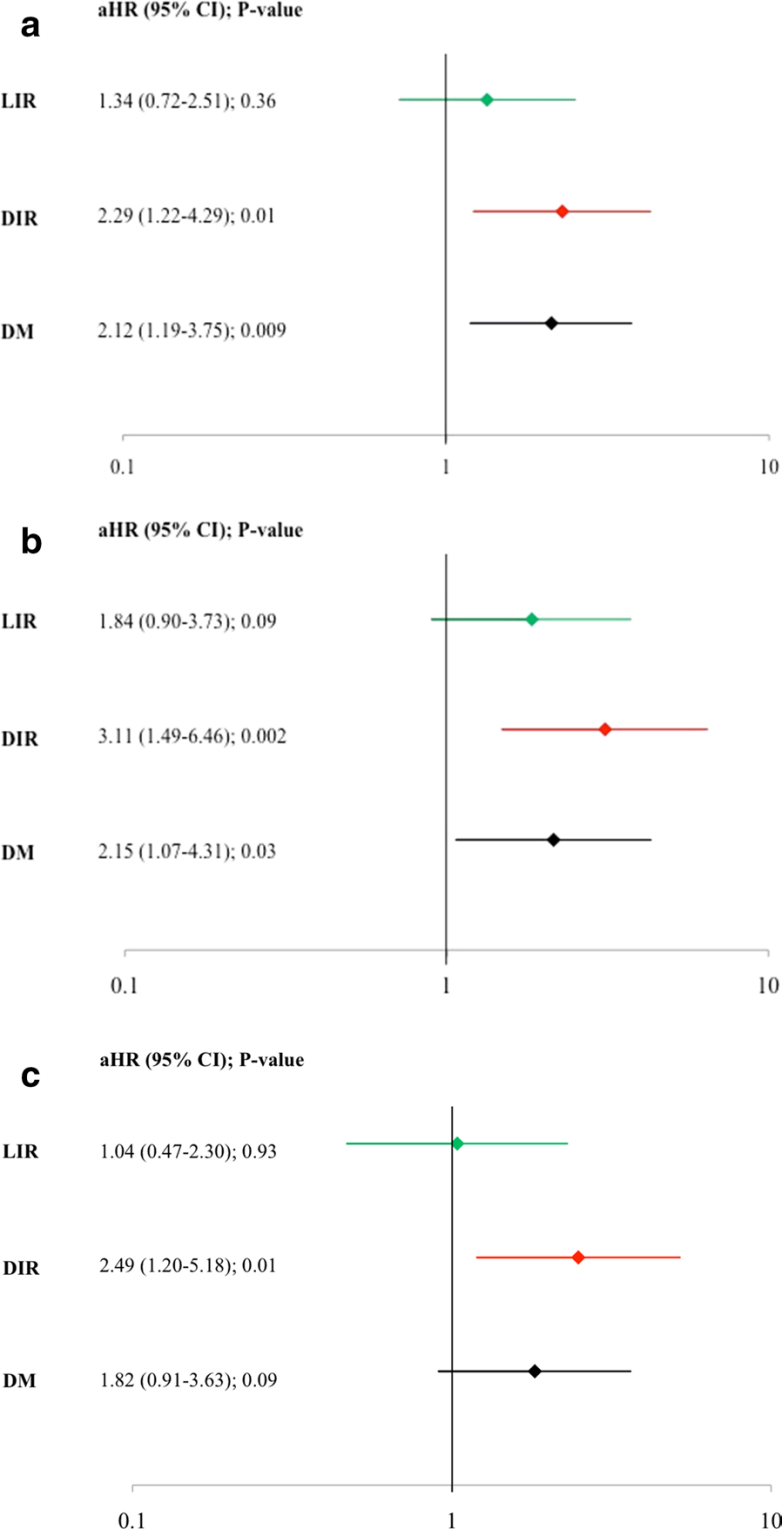
Predictors of 3-Year Outcomes. Adjusted for risk of (**a**) overall major adverse cardiac events (MACE), **b** culprit lesion MACE, and (**c**) non-culprit lesion MACE. aHR = adjusted hazard ratio; CI = confidence interval; DIR = diabetic insulin resistance; DM = diabetes mellitus; LIR = likely insulin resistant. **b** and **c** were adjusted for age, sex, use of aspirin in the preceding 7 days, and history of percutaneous coronary intervention due to the low number of events in each group

Table 3 presents 3-year clinical endpoints stratified according to HOMA-IR. Patients with DIR and DM had significantly higher crude MACE rates compared with LIR and IS (29.9%, 27.7%, 17.6%, and 15.5%, respectively, p = 0.01), which was mainly driven by higher rates of revascularization and myocardial infarction in patients with DIR and DM (Table 3 and Fig. 1). The crude MACE rates related to the culprit lesion were also higher in DIR and DM than LIR and IS patients (20.4%, 17.3%, 13.8%, and 8%, respectively, p = 0.02, Fig. 1). Definite and probable ST occurred only in patients with DIR and DM (4.7% and 3.4%, respectively, versus 0% in the LIR and IS groups, p = 0.004). Compared with IS, the adjusted risk of MACE was significantly higher for DIR and DM but not LIR (Fig. 2a). Similarly, HOMA-IR ≥ 5 was independently predictive of culprit MACE and a trend for non-culprit MACE (Fig. 2b and c).

**Table 3:** Cumulative rates of major adverse cardiac events

	Insulin sensitive (n = 234)	Likely insulin resistance (n = 109)	Diabetic insulin resistance (n = 65)	Diabetes mellitus (n = 99)	p value
Overall					
Major adverse cardiac events*^a^	15.5 (33)	17.6 (18)	29.9 (19)	27.7 (24)	0.01
Cardiac death	2.4 (5)	0 (0)	3.2 (2)	1.2 (1)	0.38
Myocardial infarction^b^	0.9 (2)	1.0 (1)	5.0 (3)	7.2 (6)	0.01
Cardiac death, cardiac arrest, or myocardial infarction	2.9 (6)	1.0 (1)	8.1 (5)	8.3 (7)	0.03
Rehospitalization due to unstable or increasing angina	14.1 (30)	16.6 (17)	25.3 (16)	22.9 (20)	0.09
Revascularization (PCI or CABG)^c^	10.9 (23)	14.7 (15)	27.1 (17)	23.5 (20)	0.003
Stent thrombosis^d^	2.0 (4)	0 (0)	4.7 (3)	4.6 (4)	0.11
Culprit lesion					
Major adverse cardiac events*^a^	8.0 (17)	13.8 (14)	20.4 (13)	17.3 (15)	0.02
Cardiac death	0 (0)	0 (0)	1.6 (1)	0 (0)	0.09
Myocardial infarction^e^	0.5 (1)	0 (0)	1.6 (1)	4.6 (4)	0.02
Cardiac death, cardiac arrest, or myocardial infarction	0.5 (1)	0 (0)	3.1 (2)	4.6 (4)	0.02
Rehospitalization due to unstable or increasing angina	8.0 (17)	13.8 (14)	19.1 (12)	13.8 (12)	0.07
Revascularization (PCI or CABG)*	6.2 (13)	10.8 (11)	17.5 (11)	15.2 (13)	0.02
Stent thrombosis^c,d^	0.0 (0)	0.0 (0)	4.7 (3)	3.4 (3)	0.004
Non-culprit lesion					
Major adverse cardiac events^a^	9.2 (19)	8.9 (9)	19.2 (12)	16.5 (14)	0.057
Cardiac death	0 (0)	0 (0)	0 (0)	0 (0)	–
Myocardial infarction	0.0 (0)	1.0 (1)	3.4 (2)	1.4 (1)	0.13
Rehospitalization due to unstable or increasing angina	9.2 (19)	7.9 (8)	17.5 (11)	15.1 (13)	0.10
Revascularization (PCI or CABG)^f^	7.8 (16)	7.9 (8)	19.2 (12)	14.1 (12)	0.03
Indeterminate events					
Major adverse cardiac events^a^	2.9 (6)	1.0 (1)	1.7 (1)	4.7 (4)	0.44
Cardiac death	2.4 (5)	0 (0)	1.7 (1)	1.2 (1)	0.46
Myocardial infarction	0.5 (1)	0 (0)	0 (0)	1.1 (1)	0.62
Rehospitalization due to unstable or increasing angina	0.5 (1)	1.0 (1)	0 (0)	2.4 (2)	0.39
Revascularization (PCI or CABG)^f^	0 (0)	0 (0)	0 (0)	0 (0)	–

^*^p < 0.03 comparing insulin sensitive (IS) with diabetic insulin resistance (DIR) and diabetes mellitus (DM); ^a^ cardiac death or arrest, myocardial infarction, rehospitalization for unstable angina, or increasing angina; ^b^p < 0.05 comparing IS and DM; ^c^p < 0.005 comparing IS with DIR and DM; ^d^definite or possible per Academic Research Consortium definition; ^e^p = 0.01 comparing IS with DM; ^f^p < 0.005 comparing IS with DIR. CABG, coronary artery bypass grafting; PCI, percutaneous coronary intervention

## Discussion

The key findings of the present analysis from the *PROSPECT* study are (i) the prevalence of DIR is high among non-DM patients with ACS, (ii) both DM and DIR estimated using the HOMA index are associated with more echolucent plaques compared with patients with normal or moderate IR, and (iii) both DIR and DM were predictive of ischemic events after ACS; DIR predicted MACE in culprit as well as non-culprit lesions and DM predicted culprit lesion MACE in this analysis. Thus, among patients with ACS, not only patients with DM but also DIR constitute high-risk subgroups.

### IR and the development/progression of atherosclerosis:

DM is associated with accelerated progression of atherosclerosis and cardiovascular disease [17] and is a widespread disorder that develops gradually from early forms of glucometabolic disturbances to clinically manifest DM [18]. While the pathophysiology of atherosclerotic plaque development is multifactorial and impacted by obesity, lipoprotein metabolism, hypeglycemia and inflammation [19–23], even early stages of impaired glucometabolism such as IR in which fasting and postprandial glucose levels remain within normal limits [4, 18], may be associated with some of the underlying mechanisms. With deterioration of insulin sensitivity, the pancreatic ß-cells respond to the elevated glucose concentration by increasing insulin production. Hyperinsulinemia, in turn, has been linked to atherosclerosis progression [24]. IR has a pro-inflammatory and pro-coagulatory effect and is associated with endothelial dysfunction [20, 25]. Supporting evidence for a complex interplay of many different factors resulting from metabolic disturbances as the basis of atherosclerosis progression was derived from a study in patients with stable coronary artery disease [26]. Low PCSK9 plasma levels were associated with a particular metabolic phenotype (low HDL cholesterol, the metabolic syndrome, obesity, insulin resistance and diabetes) [26] and a higher computed tomography angiography score which was computed by combining the extent, severity, composition, and location of atherosclerotic plaques [27]. In the present study, not only patients with DM but also those with DIR had higher levels of insulin, and fasting glucose, waist circumference, BMI and more proatherogenic lipid profile. Both hyperinsulinemia and IR have been shown to be associated with incident CAD, independent of traditional cardiovascular risk factors [28–30]. Furthermore, IR was found to be independently associated de-novo ischemic heart disease and new PCI even in subjects with normal glucose tolerance [31]. Therefore, it is not surprising, however, important to acknowledge that the proportion of patients with any level of glucometabolic disturbance in our ACS population was high.

Studies in manifest diabetes have found an association between an increase in HbA1c and HOMA-IR and coronary plaque progression [27]. In addition, increased duration of DM combined with higher HbA1c levels In obesity, acute hyperglycemia, and proatherogenic lipid profile were linked to progression of atherosclerosis and cardiovascular outcomes [21, 22, 26, 31, 32]. These patients had higher prevalence of more advanced atherosclerotic plaque features compared with patients with LIR and IS.

### IR and plaque vulnerability:

Despite extensive investigation on factors leading to plaque instability the mechanisms resulting in the development of vulnerable plaques have not been fully elucidated. One study using optical coherence tomography found an association of longer duration of DM and higher HbA1c with increased prevalence of lipid-rich plaques, TCFA, and plaque ruptures of culprit lesions in patients with AMI [32]. Another recent study suggested that an impairment of the glycocalyx and lower levels of syndecan-1 may be involved in the development of vulnerable plaques [19]. Other data suggested that plaque vulnerability is related to abnormal abdominal fat distribution, rather than with the visceral or subcutaneous fat amount alone in patients with ACS [22]. Recent investigations have also linked IR with specific IVUS features of coronary plaque vulnerability [6, 7, 33]. Iguchi et al. [7] investigated 155 consecutive patients with stable CAD and ACS using optical coherence tomography and found a strong correlation between TCFA and IR. Additionally, coronary plaques from patients in the highest tertile of IR showed higher lipid content compared with middle and lower IR tertiles [7]. In a study with a similar design but using IVUS as imaging tool, Amano et al. [6] found higher rates of lipid-rich plaque in patients with a higher degree of IR compared to those with a lower degree of IR. Mitsuhashi et al. [33] were able to show that higher insulin secretion was associated with coronary plaques with lipid content. In the present study, patients with DIR and DM showed significantly higher rates of echolucent plaques compared with LIR and IS, however, there were no differences with respect to necrotic core, calcium content, fibrous tissue, and fibrofatty tissue among the investigated groups. While echolucent plaque indicates early stages of atherosclerosis including either lipid pool in pathological intimal thickening or early necrotic core, the presence of TCFA specifies more advanced atherosclerosis [34]. Unlike previous investigations we were not able to show a correlation between TCFA and IR status. There are several possible explanations for these discrepant results. First, we investigated patients with ACS and performed IVUS analysis of the non-culprit vessels after successful PCI of the culprit lesion. Iguchi et al. [7] and Amano et al. [6] studied a wide spectrum of CAD patients including stable CAD and ACS, and only investigated culprit lesions by intracoronary imaging. Second, although IVUS was performed in all major epicardial vessels, only the first 6 to 8 cm were investigated in *PROSPECT*. Previous studies have shown that the distal portions of the coronary arteries are particularly often diseased in patients with DM [35, 36], which may also be the case in patients with DIR. Finally, the *PROSPECT* study compared with previous investigations was a prospective, multicenter study utilizing blinded core lab IVUS analysis and independent outcome data adjudication.

### Association of IR and outcomes after ACS

The present *PROSPECT* substudy demonstrated an independent association between markedly elevated HOMA-IR with future cardiovascular events. The DIR-associated risk of MACE observed in *PROSPECT* is also consistent with the literature, as a recent meta-analysis showed that HOMA-IR ≥ 5 was an independent correlate of cardiovascular events in patients without DM [37]. The observation that the adjusted risk of MACE was increased in patients with DIR but not LIR is consistent with previous research, which showed that earlier stages of hyperglycemia have a more favorable outcome compared with advanced stages of pre-DM [38, 39]. The significantly higher risk of definite/probable ST in DIR and DM compared to IS and LIR patients is in keeping with the pro-coagulatory effects of.advanced glucometabolic disturbances discussed above. However, this finding should be interpreted with caution due to the small sample size of the present study and the lack of statistical power with regards to the endpoint of ST. As such, these findings are encouraging and imply that greater attention and more comprehensive screening for subclinical glucometabolic disturbances and earlier initiation of therapy may reduce the risk of adverse cardiovascular events for patients with IR [21, 40]. Indeed, earlier trials showed a reduction in the conversion rate from pre-DM to overt DM through lifestyle modification and early initiation of medical therapy [41]. Interestingly, in the present study, DIR was associated with both culprit and non-culprit lesion MACE while DM was only associated with culprit lesion MACE. Other than a play of chance one can hypothesize whether antihypeglycemic treatment in the DM but not in DIR group has mediated these findings. These observations are particularly important considering the high prevalence of IR among patients with ACS, with a recently reported incidence of IR of 55% among non-diabetic patients presenting with ST-segment elevation myocardial infarction [42], and with HOMA-IR ≥ 2 found in 42.6% of patients without established DM in *PROSPECT*.

## Limitations

This analysis from the *PROSPECT* study has several limitations. First, as this study was a post hoc analysis of a prospective study, our findings should be interpreted as hypothesis generating rather than conclusive. Second, we used HOMA-IR as a surrogate marker of IR and not by using the gold standard method hyperinsulinemic-euglycemic clamp; however, HOMA-IR has been shown to be comparable to hyperinsulinemic-euglycemic clamp in patients with ACS [43]. Also no oral glucose tolerance test was performed in those patients not known as diabetics. Thereby the true rate of DM might be underestimated in our study. Third, as we investigated patients with ACS, we cannot rule out the influence of stress hormones, eg. catecholamines and corticosteroids, on our results [42, 44]; however, glucose tolerance has been shown to remain stable within 3 months after myocardial infarction [45]. Forth, since the *PROSPECT* study was conducted before novel antiplatelet drugs were commercially available; our analysis cannot be directly applied to patients who received novel antiplatelet drugs.

## Conclusions

IR is highly prevalent among patients with ACS. DM and advanced but not early stages of IR are independently associated with an increased risk of adverse cardiovascular events in patients after ACS. Further research is warranted to investigate the role of IR in the development and progression of coronary atherosclerosis, and the potential for therapeutic interventions specifically targeting IR.

## Data Availability

The data, analytic methods, and study materials are proprietary to the sponsor and at this time are not available to non-study participants.

## Abbreviations

DM: Diabetes mellitus
IR: Insulin resistance
ACS: Acute coronary syndromes
PCI: Percutaneous coronary intervention
VH: Virtual histology
IVUS: Intravascular ultrasound
HOMA-IR: Homeostasis model assessment
TCFA: Thin-cap fibroatheroma
MACE: Major adverse cardiovascular events
MLA: Minimum lumen area
DIR: Diabetic insulin resistance
LIR: Likely insulin resistance
IS: Insulin sensitivity
